# Gestational diabetes mellitus and children’s social-emotional development, behavioral problems, and psychological adjustment

**DOI:** 10.1038/s41390-025-04191-x

**Published:** 2025-06-10

**Authors:** Inka Mattila, Saara Nolvi, Eeva-Leena Kataja, Jetro J. Tuulari, Riikka Korja, Noora M. Scheinin, Risto Kaaja, Hasse Karlsson, Eeva Ekholm, Linnea Karlsson

**Affiliations:** 1 FinnBrain Birth Cohort Study, Turku Brain and Mind Center, Department of Clinical Medicine, University of Turku and Turku University Hospital, Turku, Finland; 2 Turku Institute for Advanced Studies, Department of Psychology and Speech-Language Pathology, University of Turku, Turku, Finland; 3 Turku Collegium for Science, Medicine and Technology, University of Turku, Turku, Finland; 4 Department of Psychiatry, University of Turku and Turku University Hospital, Turku, Finland; 5 Department of Psychology and Speech-Language Pathology, University of Turku, Turku, Finland; 6 Department of Clinical Medicine, University of Turku and Turku University Hospital, Turku, Finland; 7 Centre for Population Health Research, University of Turku and Turku University Hospital, Turku, Finland; 8 Department of Obstetrics and Gynecology, University of Turku and Turku University Hospital, Turku, Finland; 9 Department of Paediatrics and Adolescent Medicine, University of Turku and Turku University Hospital, Turku, Finland

## Abstract

**Background:**

This longitudinal cohort study investigated whether gestational diabetes mellitus (GDM) is associated with 2- and 5-year-old children’s social-emotional development and whether child sex moderates the association.

**Methods:**

FinnBrain Birth Cohort Study recruited 3808 pregnant women 2011–2015 in Finnish Turku region and Åland Islands. Their children’s social-emotional development at 2 years was measured by Brief Infant Toddler Social Emotional Assessment (BITSEA) (*N* = 1444, GDM *n* = 227) and at 5 years by Strengths and Difficulties Questionnaire (SDQ) (*N* = 1489, GDM *n* = 225). General linear models were used.

**Results:**

After adjusting for covariates, GDM was not associated with children’s social-emotional development at age 2 years. At age 5, GDM was associated with greater total behavioral difficulties (*p* = 0.025, for 1-point increase in total score *η*^2^ = 0.004). Sex-stratified analyses showed GDM was associated with greater internalizing problems in girls at 2 years (*p* = 0.044, *η*^2^ = 0.007), and greater conduct problems (*p* = 0.018, *η*^2^ = 0.007), hyperactivity/inattention (*p* = 0.001, *η*^2^ = 0.014) and total difficulties (*p* < 0.001, *η*^2^ = 0.015) in boys at 5 years. Interaction of child’s sex and GDM was observed for hyperactivity/inattention (*p* = 0.015) and total difficulties (*p* = 0.019) at 5 years.

**Conclusion:**

GDM may influence children’s social-emotional development, behavioral problems, and adjustment—and, furthermore, in a sexually dimorphic manner. Longer follow-up is needed to evaluate long-term stability of findings.

**Impact:**

- Gestational diabetes mellitus (GDM) was associated with greater total behavioral difficulties of offspring at the age of five.
- GDM was associated with greater internalizing problems in girls at 2 years
- GDM was associated with greater conduct problems, hyperactivity/inattention and total difficulties in boys at 5 years.
- GDM may influence children’s social-emotional development in a sexually dimorphic manner.
- Findings call for further study of mechanisms of children’s social-emotional development as well as shed light on GDM-related risk factors for early intervention.

## Introduction

Gestational diabetes mellitus and previously undiagnosed diabetes affect 10–25% of pregnancies worldwide.^1^ Their prevalence is also growing due to generally increasing prevalence of obesity, physical inactivity, and mothers’ age at childbirth.^2^ GDM constitutes an important public health concern because it is a known risk factor for adverse obstetric outcomes, such as shoulder dystocia, fetal macrosomia,^3^ preterm, and Cesarean delivery.^4^ In the long run, it also potentially increases the risk of both mother and child for future diabetes, obesity, and earlier onset of cardiovascular diseases.^4^

However, there is emerging epidemiological evidence for GDM’s potential adverse influence on neurodevelopmental outcomes in the offspring that may manifest in cognitive, behavioral, and social-emotional ways.^5–8^ More specifically, some extant studies have shown maternal GDM to predict poorer cognitive development and language development,^7,9^ motor development, attention-deficit hyperactivity disorder (ADHD)^8^, and autism spectrum disorders (ASD)^10^ in offspring. Nevertheless, the findings are inconclusive and mixed, with some studies suggesting a negative association,^7,11,12^ some no association,^8,13^, and some a positive association^14^ between GDM and such domains.

Likewise, there is yet little understanding of the specific mechanisms of how prenatal maternal hyperglycemia affects the *in utero* development of central nervous system (CNS). It has been suggested that GDM alters the intrauterine environment, exposing the fetus to altered levels of growth factors,^15^ and thereby cascading effects of fluctuating blood glucose levels that may cause tissue hypoxia and iron deficiency, which can alter myelination and emerging cortical connectivity, and cause local aberrations, e.g., in hippocampal neurons.^16^ Infants of diabetic mothers (IDM) might also be exposed to higher levels of oxidative stress^17^, which has been connected to ASD. IDMs might also be exposed to systemic inflammation and epigenetic changes, which have been connected to ASD and schizophrenia.^18^

While the above studies have mostly focused on the role of GDM in offspring cognitive outcomes and ASD, less studies exist on its association with social-emotional development. Further, from a methodological point of view, prior studies have tended to group mothers with pre-existing diabetes and gestational diabetes together in analyses,^6^ although typically gestational diabetes affects fetal development later than maternal pre-existing diabetes, and therefore they might have differential influences in fetus neurodevelopment.^10^ Likewise, previous studies have seldom distinguished between diet- or medication-treated GDM, although some evidence suggests medication-treated GDM may pose greater risks for adverse neurodevelopmental outcomes due to more severe hyperglycemia that might predispose fetuses to stress, chronic inflammation, and hyperinsulinemia, which in turn may interfere with fetal brain development.^19^ Prior studies also rarely provide information on adjustment of potential confounders known to affect neurodevelopment, such as maternal pre-pregnancy body-mass index (BMI), socioeconomic status (SES), mother’s age, alcohol use, smoking during pregnancy, and offspring-related covariates, such as gestational age and birth weight.^9^

Finally, recent studies indicate that GDM-related pregnancy outcomes may differ depending on fetal sex. Pregnancy of a male fetus seems to be a risk factor for GDM^20^, and male offspring of GDM mothers have higher risk for major malformations.^21^ One study showed that pregnant women with GDM carrying a male fetus have higher prevalence of insulin treatment.^22^

### Aim and hypothesis

This study aimed to investigate if maternal GDM is associated with

1. Social-emotional development at the age of 2 and
2. Behavioral problems and psychological adjustment at the age of 5 years and
3. Whether child sex moderates these associations.

Social-emotional development was measured at 2 years of child’s age using mother-reported Brief Infant-Toddler Social and Emotional Assessment (BITSEA)^23^ and at 5 years of age using mother-reported Strengths and Difficulties Questionnaire (SDQ).^24^ Based on previous literature, we hypothesized that maternal GDM would adversely influence domains of children´s social-emotional development, behavioral problems, and psychological adjustment.^7,25,26^ We further expected that there would be sex differences (interactions by sex) in these associations.^27^

## Method

### Study population

This investigation is part of The FinnBrain Birth Cohort Study, comprising prospectively enrolled pregnant women (*N* = 3808), some together with their spouses, attending the first trimester ultrasound. The study population was recruited based on personal contact with research nurses between December 2011 and April 2015 in the Turku region and the Åland Islands. All subjects gave their written informed consent. The study was conducted in accordance with the Declaration of Helsinki and approved by the Ethics Committee of the Hospital District of Southwest Finland. Of all those informed about the project, 67% participated. A detailed cohort population description is provided in Karlsson et al.^28^

Of the 3808 participating mothers, the current study included mother child dyads for those families that returned the questionnaire assessing their child’s social-emotional development and problems (BITSEA, see below) when the child was 2 years old (study population 1; *N* = 1444, singletons *N* = 1422, twins *N* = 22), and participants that returned the questionnaire assessing their child’s behavioral problems, competence and psychological adjustment when the child was 5 years old (SDQ, see below) (study population 2; *N* = 1489, singletons *N* = 1465 and twins *N* = 24).

The demographics of the mothers included age, body mass index (BMI), level of education, and parity. Age was collected by self-report questionnaires at gestational week (gw) 14. Pre-pregnancy BMI (kg/m^2^) was calculated using height (cm) and weight (kg) reported in the Finnish Medical Birth Register (FMBR) kept by the Finnish National Institute for Health and Welfare (www.thl.fi). Educational level was categorized as low (elementary school, 9 years), medium (undergraduate studies, vocational education, 9–12 years), or high (university degree or comparable, over 12 years), and was based on self-report. Information on parity (nulliparous/multiparous) information was collected primarily from FMBR and completed with questionnaire data. We also collected data from hospital records on medications (metformin, insulin) used to treat GDM when applicable. Sex (boy, girl) of the children and length of the gestation (gestation week at birth) were collected from FMBR.

Maternal depressive symptoms were assessed using the self-report questionnaire, Edinburgh Postnatal Depression Scale.^29^ EPDS is a widely used screening questionnaire for peri-partum depression, but it is also validated for prenatal screening.^30^ It contains 10 questions, which ask women to rate how they have felt during the previous 7 days. Each question is scored on a 0–3 scale, the total score thus ranging between 0–30. The cut-off score for probable depression is suggested to be 12/13 and for possible depression, 9/10.^29^ EPDS questionnaire was filled when the child was 2- and 5-years-old to control for the potential reporting bias introduced to BITSEA and SDQ, respectively.^31^

### Measures

#### Gestational diabetes mellitus (GDM)

GDM diagnosis was obtained from the FMBR. Women were screened for GDM according to the national Finnish guidelines at the time of data collection. A 75 g 2-h oral glucose tolerance test (OGTT) was performed in gestational week 26–28. The national guidelines at the time stated that OGTT should not be performed if a nulliparous woman was under 25 years old, body mass index (BMI) was below 25, and there was no family history (in parents, grandparents, siblings, or offspring) of diabetes mellitus type 2. OGTT was also not performed if primiparous women were under 40 years old, BMI below 25, or had not previously given birth to a large child (birth weight 4500 g).

Women with previous GDM, a family history of diabetes (in parents, grandparents, siblings or offspring), glucosuria, oral corticosteroid use, pre-pregnancy BMI over 35 or polycystic ovary syndrome (PCOS) were tested in early pregnancy (week 12–16) and in week 24–28 if the first OGTT was normal. The diagnostic criterion for GDM was fasting venous plasma glucose more than 5.3 (mmol/l) or 1 h blood glucose more than 10.0 or 2 h more than 8.6.^32^

#### Brief infant-toddler social and emotional assessment (BITSEA)

BITSEA is a 42-item questionnaire used to assess social–emotional and behavioral problems, delays or deficits, and competences in children aged 12–36 months.^33^ It was filled by mothers. Each item queries the child’s behavior during the previous month and is answered as ‘not true/rarely’ (0 points), ‘somewhat true/sometimes’ (1 point), or ‘very true/often’ (2 points). BITSEA consists of two scales: 1) Problem total scale (31 items, possible score range 0–62 points) and 2) Competence total scale (11 items, possible score range 0–22 points). High problem total score and/or low competence total score is indicative of possible social-emotional and behavioral problems. BITSEA items can also be classified as measuring 5 different areas of social-emotional development, as well as specific red flag items. Some of the items are used for assessing more than one area. The five areas are: externalizing problems (6 items), internalizing problems (8 items), dysregulation problems (8 items), competence items (11 items), autism spectrum disorder (ASD) (11 items).^33^

Psychometric properties, i.e., internal consistency, test–retest, and inter-rater reliability of BITSEA have been shown to be good in original^33^ and replication studies.^34^ In our samples, Cronbach’s alpha varied from poor (0.50–0.60) in competence and internalizing scales to marginal (0.60–0.70) in problem and externalizing scale scores. These figures correspond with reports by Alakortes et al.^35^ and have been considered adequate in the context of general population assessment of child social-emotional development by parental report. In the present study, BITSEA questionnaire was answered on a paper questionnaire and returned by mail or filled online (respondent preference) upon the child turning 2 years old.

#### Strengths and difficulties questionnaire (SDQ)

The SDQ is a brief measure of prosocial behavior and psychopathology of children aged 3–16 years.^24^ It was filled by mothers at the child age of 5 years. SDQ comprises five subscales that yield scores for emotional symptoms, conduct problems, hyperactivity/inattention, peer relations problems, and prosocial behavior. Each subscale consists of five items. Some items are positively and some negatively formulated. Each item is evaluated on a 3-point Likert scale as “not true,” “somewhat true,” or “certainly true”. Responses are scored 0–2 for negatively formulated items and inversely 2–0 for positively formulated items. Subscale total scores are computed by summing the individual scale items (range 0–10). Higher scores on the Prosocial behavior subscale indicate better prosocial competence, whereas higher scores on the other four subscales indicate greater social-emotional difficulties. A Total difficulty score was generated by summing the subscale scores of Emotional symptoms, Conduct problems, Hyperactivity/Inattention, and Peer problems (range 0–40).^24^

Psychometric properties, i.e., internal consistency, test–retest, and inter-rater reliability, as well as criterion validity of SDQ have been shown to be excellent in original^24^ and replication studies in the Finnish population.^36^

#### Covariates

Investigated covariates were selected based on prior literature of key factors known to be associated with GDM and/or social-emotional development, thus potentially confounding the association between the independent and dependent variables. The covariates included in the final model comprised *Child sex* (Boy or Girl),^35^ maternal *Education* (Low, Middle or High)^37^ and child birth weight adjusted for gestational age *SGAC* (Small for GA, Appropriate for GA, Large for GA),^38^ *Smoking* (no smoking, smoked in early pregnancy, smoked throughout pregnancy, smoked in early pregnancy and no information on further smoking).^39^ Maternal *BMI* (kg/m2),^40^ *EPDS* (measured when the child was 2 years old at BITSEA assessment, and in case of SDQ, when the child was 5 years old),^31^ *Parity* (nulliparous, others)^41^, and *Age* (years, measured at childbirth).^42^

Additional covariates that were considered included alcohol use,^43^ income level^8^, and relationship status^44^ that were investigated as potential confounders but not included in the final model. This was because the variance of alcohol use and relationship status was extremely low in our population, and instead of income level, we used the level of education as an indicator of socioeconomic status (SES). We also collected data on drug abuse during pregnancy, but there were no reported drug abusers in our study population, so it was not used as a confounder.

### Statistical analyses

To determine whether the covariate candidates mentioned above ought to be included in the multivariate model, descriptive statical analyses were performed. For the associations between categorical variables, Chi Squared Test was performed;, between continuous and categorical variables, *t*-test was performed (with the exception of EPDS having skewed distribution and subsequently Mann–Whitney test was applied); and between two continuous variables, Pearson correlation was used.

General linear model was used for the analyses. The main independent variable of interest was the dichotomous GDM variable (yes/no). The dependent variable was one of the five BITSEA subscales (continuous) at the child age of 2 years and in SDQ, one of the five SDQ subscales (continuous) at the child age of 5 years.

The associations between independent variable (GDM) and dependent variable (each of the five BITSEA subscales, i.e., Autism, Competence, Internalizing, Externalizing, and Problem / each of the five SDQ subscales, i.e., emotional symptoms, conduct problems, hyperactivity/inattention, peer relations problems, and prosocial behavior) were investigated using regression models of the following form:

$${BITSEA\; subscale}\,=\, 	{GDM}+{Child\; sex}+{BMI}+{EPDS}\left(2{years}\right)+{MaternalAge}\\ 	 +{Education}+{SGAC}+{Smoking}+{Parity}$$

and

$${SDQ\; subscale}= 	 \, {GDM}+{Child\; sex}+{BMI}+{EPDS}\left(5{years}\right)+{MaternalAge}\\ 	 +{Education}+{SGAC}+{Smoking} +{Parity}$$

where the response variable *BITSEA subscale/SDQ subscale* meant one of the BITSEA subscale scores /SDQ subscale scores. *GDM* and *Child sex* were binary predictor variables, maternal *Education* and child *SGAC* were three-class predictor variables, and *Smoking* was a four-class predictor variable. Maternal *BMI*, *EPDS*, *Parity*, and *Age* are continuous predictor variables.

Furthermore, sex differences in these associations were investigated by also including the GDM *×* Child sex interaction in each model, i.e., by using regression models with the following structure:

$${BITSEA\; subscale}=\,	 {GDM}+{Child\; sex}+{GDM}\times {Child\; sex}+{BMI} \\ 	+{EPDS}+{Age}+{Smoking}+{Education}+{Parity}+{SGAC}$$

$${SDQ\; subscale}=\,	 {GDM}+{Child\; sex}+{GDM}\times {Child\; sex}+{BMI} \\ 	 +{EPDS}+{Age}+{Smoking}+{Education}+{Parity}+{SGAC}$$

The data was then stratified to two subsets by child sex in order to find out whether the above relationships would differ depending on child sex. General linear model analyses were conducted in a similar fashion as described above:

$${BITSEA\; subscale}/{SDQ\; subscale}=\,	 {GDM}+{BMI}+{EPDS}\,2{years}/{EPDS}\,5{years} \\ 	+{Age}+{Smoking}+{Education} \\ 	+{Parity}+{SGAC}$$

In cases of missing values of the individual BITSEA/SDQ items, these were imputed with the mean value of the corresponding BITSEA/SDQ subscale. According to BITSEA guidelines, the maximum amount of allowed missing values in BITSEA was 4/31 in the problem and 1/11 in the competence domain. The maximum amount of allowed missing values in SDQ subscales was 2/5 in each domain.

IBM SPSS Statistics 27 was used to analyze data.

## Results

### Description of the study populations

Study population 1 consisted of the 1444 children whose mothers returned the BITSEA questionnaire out of the 3808 who received it initially (i.e., 37.9% response rate). The final study population comprised 1422 singletons and 22 twins, 227 of which were cases (IDM) and 1198 of which were controls. The characteristics of the final participating cohort mothers and children are reported in Table 1. The mothers who returned the questionnaire were on average one year older (30.7 vs 29.7 years old), more highly educated (42.5% vs 25.8% had higher education), had more frequently a GDM diagnosis (15.9% vs 14.9%) and were more likely to be nulliparous (53% vs 47%), compared to mothers who did not return the BITSEA questionnaire.

**Table 1:** Descriptives.

	Gestational diabetes mellitus		Control		All	
	BITSEA^a^ *N* = 227 (15.9%) Mean (SD/%)	SDQ^b^ *N* = 225(15.3) mean (SD/%)	BITSEA^a^ *N* = 1198 (84.1%) mean (SD/%)	SDQ^b^ *N* = 1242 (83.5%) mean (SD/%)	BITSEA^a^ *N* = 1444 mean (SD/%)	SDQ^b^ *N* = 1488 mean (SD/%)
Age at delivery	32.3 (4.4)	32.1 (4.2)	30.1 (4.3)	30.1 (4.2)	31.1 (4.3)	31.0 (4.3)
Child’s sex (girl/boy)	101 (44.5%) / 126 (55.5%)	96 (42.7%)/ 129 (57.3%)	561 (46.8%) /637 (53.2%)	572 (46.1%)/ 670 (53.9%)	662 (46.5%)/763 (53.5%)	668 (45.5%)/799 (54.5%)
Gestation weeks at delivery	39.4 (1.7)	39.4 (1.7)	39.8 (1.6)	39.8 (1.6)	39.8 (1.7)	39.8 (1.6)
BMI before pregnancy	28.1(6.8)	27.8 (6.5)	23.8 (4.2)	23.7 (4.1)	24.5 (4.9)	24.4 (4.7)
Education						
low (9 years)	70 (32.1%)	66 (31.0%)	316 (27.6%)	334 (28.0%)	386 (28.3%)	400 (28.5%)
medium (9–12 years)	65 (29.8%)	63 (29.6%)	332 (29.0%)	358 (30.1%)	397 (29.1%)	421 (30.0%)
high (over 12 years)	83 (38.1%)	84 (39.4%)	496 (43.4%)	499 (41.9%)	579 (42.5%)	583 (41.5%)
all	218	213	1144	1191	1363	1404
Relationship status						
married/domestic partnership	212(99.1%)	208 (98.6%)	1102 (98.5%)	1139 (98.4%)	1314 (98.6%)	1347 (98.4%)
others	2 (0.1%)	3 (1.4%)	17 (1.5%)	19 (1.6%)	19 (1.4%)	22 (1.6%)
all	214	211	1119	1158	1333	1369
Parity						
nulliparous	120 (52.9%)	136 (60.4%)	644 (53.8%)	651 (56.6%)	764 (53.6%)	787 (53.6%)
others	107 (47.1%)	89 (39.6%)	554 (46.2%)	591 (51.3%)	661 (46.4%)	680 (46.4%)
Income level (€)						
1500€ or lower	85 (38.8%)	77 (36.0%)	387 (33.9%)	407 (34.3%)	472 (34.7%)	484 (34.5%)
1501–2500	108 (49.3%)	119 (55.6%)	614 (53.8%)	640 (53.9%)	722 (53.1%)	759 (54.2%)
2501–3500	25 (11.4%)	17 (7.9%)	118 (10.3%)	119 (10.0%)	143 (10.5%)	136 (9.7%)
3500-	1 (0.5%)	1 (0.5%)	22 (1.9%)	21 (1.8%)	23 (1.7%)	22 (1.6%)
all					1360	1401
Smoking						
At the beginning of pregnancy (before knowing)	16 (7.0%)	13 (5.8%)	90 (7.5%)	100 (8.1%)	106	113 (7.7%)
At the end of the pregnancy	16 (7.0%)	43 (3.5%)	43 (3.6%)	13 (5.8%)	59	56 (3.8%)
No	195 (85.9%)	199 (88.4%)	1065 (88.9%)	1099 (88.5%)	1260	1298 (88.5%)
Size for gestational age (GA)						
small for GA	3 (1.3%)	5 (2.2%)	33 (2.7%)	36 (2.9%)	36 (2.5%)	41 (2.8%)
appropriate for GA	213 (93.8%)	212 (94.2%)	1140 (95.2%)	1181 (95.1%)	1353 (94.9%)	1393 (95.0%)
large for GA	11 (4.8%)	8 (3.6%)	25 (2.1%)	25 (2.0%)	36 (2.5%)	33 (2.2%)
all	227	225	1198	1242	1425	1467

*BITSEA* Brief Infant Toddler Social Emotional Assessment, *SDQ* Strenghts and Difficulties Questionnaire, *BMI* body mass index, *EPDS* eEdinburgh Postnatal DepressionsScale, *GA* gestational age.

^a^Child age of 2 years, Study population 1.

^b^Child age of 5 years, Study population 2.

Study population 2 included those 1489 children whose mothers returned the SDQ questionnaire out of the 3839 (38.8% response rate) to whom it was sent initially. Of these 1489 mother-child dyads returning the SDQ questionnaire, 1113 (74.7%) had also returned the BITSEA questionnaire. There were 1465 singletons and 24 twins in this final sample, of which cases comprised 225 and controls comprised 1242. The characteristics of the final participating cohort mothers and children are reported in Table 1. The mothers who returned the SDQ questionnaire were, on average, older (31.0 vs. 29.7 years old), were more likely to have higher education (41.5% vs.26.2%), and be nulliparous (53.6% vs. 47.1%) as compared to the mothers who did not return the SDQ questionnaire.

### Associations between independent variables and potential confounding factors

In study population 1, GDM was associated with higher maternal age (*r* = 0.114, *p* < 0.001), higher SGAC (*χ*^2^ = 7.337, df = 2, *p* = 0.03), higher maternal BMI (*r* = 0.312, *p* < 0.001), and lower gestational weeks at delivery (*r* = −0.094, *p* < 0.001). There was no evidence that GDM would be related to maternal smoking, drug use, alcohol use, income level, relationship status, parity, EPDS symptom score, or the level of education (all *p* values > 0.05).

In study population 2, GDM was associated with higher maternal age (*r* = 0.107, *p* < 0.001), BMI (*r* = 0.307, *p* < 0.001), gestational weeks at delivery (*r* = −0.203, *p* < 0.001) and parity (*χ*^2^ = 16.388, df < 06, *p* = 0.012).

### Association between dependent variables and potential confounding factors

Multiple statistically significant associations were observed between dependent variables and potential confounding variables in both study populations 1 and 2. Based on both theoretical considerations and prior empirical findings, selection of potential confounders was deliberately inclusive. Thus, adjustments in the final analyses were ultimately made for SGAC, sex of the child, maternal age, level of education, BMI, smoking, and parity, as well as EPDS total score at the child age of 2 years (in the case of BITSEA analyses) or 5 years (in the case of SDQ analyses).

### Maternal GDM and offspring BITSEA and SDQ scores

#### Child outcomes at 2 years

In bivariate analyses, maternal GDM was not associated with offspring BITSEA subscale scores. After adjusting for covariates, BITSEA subscale scores were not associated with maternal GDM status either (Table 2). Interaction analyses (child sex by maternal GDM) indicated no statistically significant sex differences (*p*-values for each BITSEA subscale x child’s sex > 0.41).

**Table 2:** General linear model of gestational diabetes mellitus as a predictor of BITSEA (Brief Infant Toddler Social Emotional Assessment) subscales after adjusting for maternal body mass index, age, education, maternal depressive symptoms (EPDS), smoking, and child’s sex, birth weight (adjusted for gestational age), and parity.

	*B*	95% CI (lower bound, upper bound)	*t*	*p*
BITSEA subscale				
Autism	0.318	−0.060, 0.696	1.890	0.099
Externalizing	0.030	−0.278, 0.338,	0.191	0.848
Internalizing	0.239	−0.155, 0.632,	1.191	0.239
Competence	−0.241	−0.626, 0.143	−1.231	0.218
Problem	0.429	−0.238, 1.095	1.262	0.207
BITSEA boys subscale				
Autism	0.238	−0.286, 0.762	0.892	0.373
Externalizing	0.075	−0.367, 0.517	0.332	0.740
Internalizing	−0.058	−0.555, 0.440	−0.228	0.819
Competence	−0.316	−0.857, 0.226	−1.144	0.253
Problem	0.118	−0.761, 0.997	0.263	0.792
BITSEA girls subscale				
Autism	0.462	−0.092, 1.016,	1.637	0.102
Externalizing	−0.052	−0.480, 0.377	−0.236	0.813
Internalizing	0.655	0.017, 1.293	2.017	0.044
Competence	−0.226	−0.771, 0.319,	−0.816	0.415
Problem	0.843	−0.192, 1.878	1.600	0.110
SDQ subscale				
F1 Emotional symptoms	0.091	−0.113, 0.295	0.877	0.381
F2 Conduct problems	0.281	−0.025, 0.586	1.801	0.072
F3 Hyperactivity/inattention	0.342	−0.009, 0.693	1.910	0.056
F4 Peer relations problems	0.125	−0.105, 0.355	1.064	0.288
F5 Prosocial behavior	0.030	−0.265, 0.324	0.197	0.843
Total difficulties score	0.838	0.107, 1.569	2.250	0.025
SDQ boys subscale				
F1 Emotional symptoms	0.120	−0.149, 0.390	0.878	0.380
F2 Conduct problems	0.492	0.083, 0.901	2.362	0.018
F3 Hyperactivity/inattention	0.781	0.311, 1.250	3.263	0.001
F4 Peer relations problems	0.319	−0.015, 0.653	1.878	0.061
F5 Prosocial behavior	0.053	−0.351, 0.457	0.257	0.797
Total difficulties score	1.713	0.716, 2.710	3.372	<0.001
SDQ girls subscale				
F1 Emotional symptoms	0.066	−0.251, 0.383	0.411	0.681
F2 Conduct problems	0.023	−0.443, 0.490	0.098	0.922
F3 Hyperactivity/inattention	−0.194	−0.727, 0.339	−0.716	0.475
F4 Peer relations problems	−0.124	−0.425, 0.178	−0.804	0.422
F5 Prosocial behavior	−0.027	−0.462, 0.407	−0.122	0.903
Total difficulties score	−0.228	−1.302, 0.845	−0.417	0.677

*BITSEA* brief infant-toddler social emotional assessment, *SDQ* strengths and difficulties questionnaire, *EPDS* edinburgh postnatal depression scale.

#### Child outcomes at 5 years

In bivariate analyses, GDM was associated with greater SDQ conduct problems (*B* = 0.323, *t* = 2.220, CI = 0.038, 0.608, *p* = 0.027), hyperactivity/inattention (*B* = 0.428, *t* = 2.530, CI = 0.096, 0.760, *p* = 0.012), and total difficulties score (*B* = 0.920, *t* = 2.537, CI = 0.209, 1.632, *p* = 0.011). After adjusting for covariates, a statistically significant association remained for the SDQ total difficulties score (*B* = 0.838, *t* = 2.250, CI = 0,107, 1.569 partial *η*^2^ = 0.004, *p* = 0.025). For the remaining subscales see Table 2. for the *p* values.

The inclusion of GDM treatment type (only diet/ + Metformin/ + Insulin) into the models did not alter the results (data not shown).

### Interaction analyses

There were statistically significant interactions at 5-year-olds (child sex by maternal GDM) in predicting child hyperactivity/inattention (*B* = 0.832, *t* = 2.436, CI = 0.162, 1.502, *p* = 0.015) and total difficulties score (*B* = 1.663, *t* = 2.339, CI = 0.268, 3.058, *p* = 0.019) with boys showing more total difficulties and hyperactivity/inattention. The *p* values for the remaining SDQ subscales x child’s sex were as follows: emotional symptoms 0.849, conduct problems 0.094, peer relations problems 0.1, and prosocial behavior 0.871.

### Stratified analyses

Analyses stratified by child’s sex showed that maternal GDM was associated with BITSEA internalizing subscale (*B* = 0.655, *t* = 2.017, CI = 0.017, 1.293, partial *η*^2^ = 0.007, *p* = 0.044) in girls. Among boys, maternal GDM was associated with SDQ subscale of conduct problems (*B* = 4.92, *t* = 2.362, CI = 0.083, 0.901, partial *η*^2^ = 0.007, *p* = 0.018), hyperactivity/inattention (*B* = 0.781, *t* = 3.263, CI = 0.311, 1.250, partial *η*^2^ = 0.014, *p* = 0.001) and total difficulties score (*B* = 1.713, *t* = 3.372, CI = 0.716, 2.710, partial *η*^2^ = 0.015, *p* < 0.001).

## Discussion

The aim of this study was to investigate whether maternal GDM influences children’s social-emotional development, behavioral problems, and psychological adjustment at 2 and 5 years of age. We found very little evidence for maternal GDM explaining the variance in social-emotional development in 2-year-olds. However, we observed that behavioral problems and psychological adjustment in 5-year-olds were associated with exposure to GDM, although the effect sizes were low. Further, male and female offspring were differentially influenced by maternal GDM. Adjusting the model with GDM treatment type (which can also be viewed as a proxy for GDM severity) did not change the results.

First, the general lack of associations with social-emotional characteristics at 2 years of age is in line with the previous inconclusive findings on GDM and neurodevelopmental correlates that have previously mainly focused on cognitive outcomes and ASD.^6^ Nevertheless, it might be noted that prior studies have typically included both pregestational and gestational diabetes mellitus cases, as well as often been considerably smaller in sample size. In one study with a larger sample (221 infants born to mothers with GDM and 2612 controls) that also differentiated between types of diabetes, Dionne et al.^11^ found GDM to predict adverse effects on language development as measured in terms of vocabulary and grammar performance at 18 and 30 months, after adjusting for child’s sex, gestational age, birth weight, Apgar score, gestational hypertension, alcohol use and smoking in pregnancy. Besides the different outcome focus (i.e., language development), this study did not adjust for maternal BMI, unlike our study.

Indeed, the lack of association between GDM and social-emotional functioning in the present study among 2-year-old children may reflect the characteristics of the general population sample, where variance regarding prominent psychosocial problems may be low among young children. Thus, the subsequent low base rate of some item scorings among toddlers may also be reflected in the reported modest alphas in BITSEA. While it is possible that the null finding among 2-year-olds is a true observation, it also remains a possibility that any existing associations were not detected per the limitations of questionnaire assessment of our outcome in this population. Another, not mutually exclusive, potential explanation is that as children get older, the effects of GDM and/or their interaction with environment may become more pronounced and detectable. For example, Nomura et al.^8^ found GDM to predict poorer language, visuospatial, and memory scores in 3–4-year-olds (21 GDM children and 191 control subjects). Speaking to the importance of environment, they also reported a significant interaction effect between low socio-economic status and GDM.^8^

In contrast to the findings at 2 years of age, and in line with our hypothesis, GDM was relatively consistently associated with several behavioral problems and psychological adjustment in the age group of 5-year-olds, and this association with total difficulties remained significant after adjusting for confounders. This finding aligns with previous studies on other outcome domains, showing GDM to be associated with poorer cognitive and especially language development. However, a limitation of this earlier research, in addition to small sample sizes, is that it has generally not tended to adjust (or has failed to report) for even well-known confounding factors, such as maternal BMI or level of education.^6^ A study comprising only 23 GDM mothers did adjust for confounding factors (total *n* = 5282)^12^ and found GDM to predict negative verbal IQ at 8 years’ age after adjusting for maternal age, BMI, SES, level of education, lifestyle behaviors, parity, duration of breastfeeding, birth weight, gestational age and sex. Yet the present study is, to our knowledge, the first one to investigate and report an interaction effect of GDM with child sex on behavioral problems and psychological adjustment, showing an increased risk of hyperactivity/inattention, conduct problems, and total difficulties in boys.

Our finding also differs from what, to our knowledge, is the only other study to investigate the effect of GDM on social-emotional development and behavioral problems, and psychological adjustment. A study was conducted in Greece by Daraki et al.^45^ on 4-year-olds with 772 mother and child pairs. After adjusting for child sex, study assessor and quality of assessment, maternal BMI, maternal age, maternal origin, maternal education level, parity, and maternal smoking during pregnancy, they found that GDM was not associated with SDQ scores (nor McCarthy's Scales of Children’s Abilities). One reason for the difference in our findings might be explained by the smaller number of mothers with GDM diagnosis (*n* = 55) in the Greek study.

Recent evidence points to fetal sex related GDM effects on offspring, such as neonatal adiposity and metabolic dysfunction. For instance, Bahado-Singh et al.^46^ investigated whether treatment (dietary intervention, self-monitoring of blood glucose, and insulin therapy, if necessary) of GDM affected maternal and perinatal outcomes differentially on the basis of fetal sex. They found that male fetuses were more likely to have a lower birth weight and less neonatal mass when treated for GDM, whereas these differences were not significant among female offspring. One possible explanation may be that male fetuses are suggested to be more susceptible to oxidative stress^47^ (OS), which would be increased in GDM by elevated maternal blood and placental tissue glucose,^48^ known to have an important role in lipogenesis and fat accumulation. Correspondingly, male fetuses appear to benefit more from treatment reducing OS.^46^ Another possible mechanism for interactions between maternal GDM and fetal sex is that placental adaptive responses seem more pronounced in female fetuses.^49,50^ The significance of these processes for social-emotional, behavioral, and psychological outcomes in the context of GDM exposure has been nevertheless very scarcely studied and remains little understood. For example, Fraser et al.^26^ investigated the association of maternal GDM and IQ (measured in mandatory conscription examination) at 18 years old (*n* = 664,871) singleton men. While they found GDM to be associated with lower IQ in non-siblings, no association was noted among the siblings.

This suggests that the associations between GDM and at least some offspring outcomes in adults might be explained by shared familial characteristics or individual differences in vulnerability rather than in utero environment.

### Limitations

The strengths of the present study include a prospective pregnancy cohort study design, a large sample size, two measurement points for outcomes at developmentally different ages, and well-established outcome measures. We were able to use a well-defined GDM phenotype, exclude previously diagnosed diabetes, and analyze the effect of GDM treatment type (diet/insulin/metformin), although the number of insulin-treated gestational diabetics was low. Among the main strengths compared to prior research, besides the large sample size, is the comprehensive information on many well-known confounders.

Nevertheless, some limitations also exist. First, our study population was ethnically homogeneous, mainly Finnish/Caucasian. Given that previous studies have shown ethnic differences in GDM prevalence, severity, and pregnancy outcomes, the present findings might not apply to other ethnic groups.^51^ Second, although we incorporated extensive information on potential social and environmental factors associated with child neurodevelopment, residual confounding may still occur.

## Conclusion

We found GDM to be associated with increased behavioral and adjustment problems at the age of 5 years, with the association especially pronounced among boys. No associations were observed between GDM and social-emotional and behavioral problems at the age of 2 years, with the exception of more internalizing problems in girls in sex-stratified analyses. In the future, research is needed to investigate the possible influences of GDM treatment on these offspring outcomes, as well as extend the follow-up to evaluate the long-term stability of the observed influences.

## Data Availability

The datasets presented in this article cannot be made fully openly available based on Finland’s national personal data legislation and FinnBrain cohort’s ethics regulations. Access to the datasets can be requested via collaboration and by contacting the Board of the FinnBrain Birth Cohort Study (PI Linnea Karlsson, linnea.karlsson@utu.fi). International and domestic collaboration is encouraged and inherent in the project, and the ultimate goal is that the data will be available as easily as possible for the research community.
